# Isolated Musculocutaneous Neuropathy Secondary to an Immune-Mediated Brachial Plexopathy: A Case Report of a Rare Phenotype With a Side Note on Patterns of Weakness and an Update on Nerve Pathology

**DOI:** 10.7759/cureus.10267

**Published:** 2020-09-05

**Authors:** Hassan Kesserwani

**Affiliations:** 1 Neurology, Flowers Medical Group, Dothan, USA

**Keywords:** peripheral nerve disorders, brachial plexopathy

## Abstract

We describe a 50-year-old man who developed a low-frequency pattern of weakness, musculocutaneous neuropathy, with weakness of the biceps, coracobrachialis and brachialis in an immune-mediated brachial plexopathy. The aim of this article is to highlight both the low- and high-frequency patterns of weakness of the immune-mediated brachial plexopathies, and we focus on the patterns of recognition. We then segue into the pathology of the immune-mediated plexopathies and highlight the recent spectacular magnetic resonance imaging studies that demonstrate hourglass constrictions of peripheral nerves outside of the brachial plexus in afflicted patients. This opens up a window for the exciting possibility of neurolysis of constricted nerves in patients who have not responded adequately to immunotherapy.

## Introduction

The musculocutaneous nerve (MCN) arises from the lateral cord of the brachial plexus, deriving from the C5, C6 and C7 nerve roots. The principal branch of the MCN penetrates the coracobrachialis muscle with a mean distance from the coracoid process to the point of penetration of approximately 6 centimeters (cm) [1]. It supplies the biceps, brachioradialis and coracobrachialis (BBC) muscles. The BBC muscles are the three anterior muscles of the upper arm. The biceps is double-headed with a dual action: flexion of the elbow and supination of the forearm. The brachialis muscle flexes the elbow joint. It lies deeper than the biceps and it makes up part of the floor of the cubital fossa. The brachialis muscle is the prime mover of elbow flexion generating 75% of the power of elbow flexion. The brachialis muscle is innervated by the musculocutaneous nerve and in 70-80% of cases the muscle also receives innervation from the radial nerve, C5-T1. The coracobrachialis flexes the upper arm at the shoulder joint and draws the humerus toward the torso, hence adducting the upper arm. The terminal branch, the lateral cutaneous nerve of the forearm is purely sensory and supplies sensation to the lateral aspect of the forearm from the elbow to the wrist. It is also known as the musculocutaneous sensory nerve. 

The MCN can be injured followed strenuous muscle activity by neurapraxia or axontmesis as it passes through the coracobrachialis in the upper arm [2]. More commonly, entrapment of the musculocutaneous sensory nerve can occur below the biceps aponeurosis after strenuous elbow extension or forearm pronation. This usually presents with anterolateral burning of the forearm, usually necessitating nerve decompression [3]. MCN injury has also been described with blunt trauma such as shoulder dislocation [4]. It has also been described as a manifestation of neuralgic amyotrophy, also known as immune-mediated brachial plexopathy [5]. There are also other rarer anecdotal reports, such as a proximal humeral exostosis leading to an isolated musculocutaneous neuropathy [6]. 

Immune-mediated brachial plexopathy is also known as neuralgic amyotrophy, brachial neuritis, or Parsonage Turner syndrome. It is usually of sudden onset, associated with severe pain of the shoulder girdle with weakness and atrophy of the limb, mostly proximal, with variable sensory symptoms. It is occasionally associated with weight loss and in some instances an antecedent viral illness or vaccination. It is usually monophasic, with a nadir within weeks and slow resolution over six to 12 months, even up to three years [7]. Incomplete recovery is common and response to immunotherapy variable. In the rare instances where a brachial plexus nerve biopsy has been reported, a multifocal endoneurial and perineurial mononuclear inflammatory cell infiltrate has been noted but without fibrinoid necrosis to suggest necrotizing vasculitis [8].

We will expand on the patterns of weakness of the immune-mediated brachial plexopathies during the discussion section. This is an exercise in anatomic localization and pattern recognition. However, a listing in order of frequency includes the upper trunk plexopathy, the so-called "shoulder girdle syndrome", by far the most common pattern of weakness. This is followed by a lower trunk pattern with predominant hand weakness. A middle trunk plexopathy has never been reported in isolation. An isolated posterior interosseous nerve injury with finger drop has been observed rarely. The rest of the patterns are of low frequency and these include a long thoracic nerve palsy with "winging of the scapula", an isolated phrenic nerve palsy with dyspnea and hemi-diaphragm elevation, an MCN neuritis with a BBC pattern of weakness and an anterior interosseus neuritis with weakness of pinching. These low-frequency patterns can occur with the higher frequency patterns, as a mixed combination [7].

A right-handed 50-year-old man presented with a two-week history of severe left shoulder pain followed by a BBC pattern of weakness. The left biceps atrophy was striking. We demonstrate the classic findings of BBC denervation on electromyography (EMG) and an absent musculocutaneous sensory nerve amplitude on nerve conduction study (NCS), implicating the MCN. The patient reached a plateau, as expected with an immune-mediated brachial plexopathy, after two weeks. He was treated with weekly intravenous 500 milligrams (mg) pulse solumedrol for one month, with little improvement of power. Intravenous immunoglobulin is planned on an empiric basis. In the discussion segment, we outline the clinical characteristics of the patterns of weakness seen in immune-mediated brachial plexopathies. We also focus on the main immunopathology study, and highlight the recent amazing findings on radiologic studies of hourglass constrictions of peripheral nerves outside of the brachial plexus. We will closely monitor our patient's progress with respect to this new development in the field, as demonstration of hourglass constriction on magnetic resonance imaging studies opens up a window for surgical neurolysis. We will explore this new spectacular development in the discussion segment.

## Case presentation

We present the case of a right-handed 50-year-old man who presented with a one-week history of excruciating and debilitating left shoulder and left upper arm pain. This pain had subsided by two weeks and was accompanied by burning pain and a feeling of numbness over the lateral aspect of his forearm. He developed left biceps atrophy that was quite striking and advanced by six weeks. He denied any neck pain or left-hand weakness. He was able to feed himself but he had problems lifting heavier objects with the left arm. He denied any weight loss. 

His past medical history was significant for low-grade hyperlipidemia, well-controlled hypertension, well-controlled diabetes and psoriatic arthritis requiring immunotherapy. His medications included lisinopril, atorvastatin, metformin and monthly subcutaneous injections of an interleukin-17 antagonist, secukinumab, for the last two years. On examination his blood pressure (BP) was 120/60 with a pulse of 54. His height is 5 foot and 10 inches with a weight of 229 pounds and body mass index (BMI) of 32.9. The pertinent findings will be listed on examination. 

On auscultation of the lungs and percussion of the chest there was no suggestion of an elevated hemi-diaphragm. His breathing was comfortable in the supine position. Of note, his cranial nerve examination was entirely normal. We need to mention that he did not manifest a Horner's syndrome or facial weakness. Right upper and bilateral lower extremity power was entirely normal with the medical research council grading (MRC), 5/5 for all the major muscle groups. Left upper extremity power is graded below (Table 1).

**Table 1:** Left upper extremity muscular power testing: a pattern emerges; weakness of biceps, brachialis and coracobrachialis

LEFT UPPER EXTREMITY MUSCLE	POWER GRADING USING MRC SCALE (1-5)
Supraspinatus	5
Deltoid	5
Biceps	3
Brachioradialis	5
Brachialis	4
Coracobrachialis	4
Triceps	5
Pronator teres	5
Supinator	5
Extensor digitorum communis	5
Extensor carpi radialis longus	5
Flexor carpi ulnaris	5
Flexor carpi radialis	5
Extensor pollicis longus	5
Flexor pollicis longus	5
Interossei	5
Abductor pollicis brevis	5
Adductor pollicis	5
Sublimis and flexor digitorum profundus to all 4 digits	5 and 5

**Figure 1:**
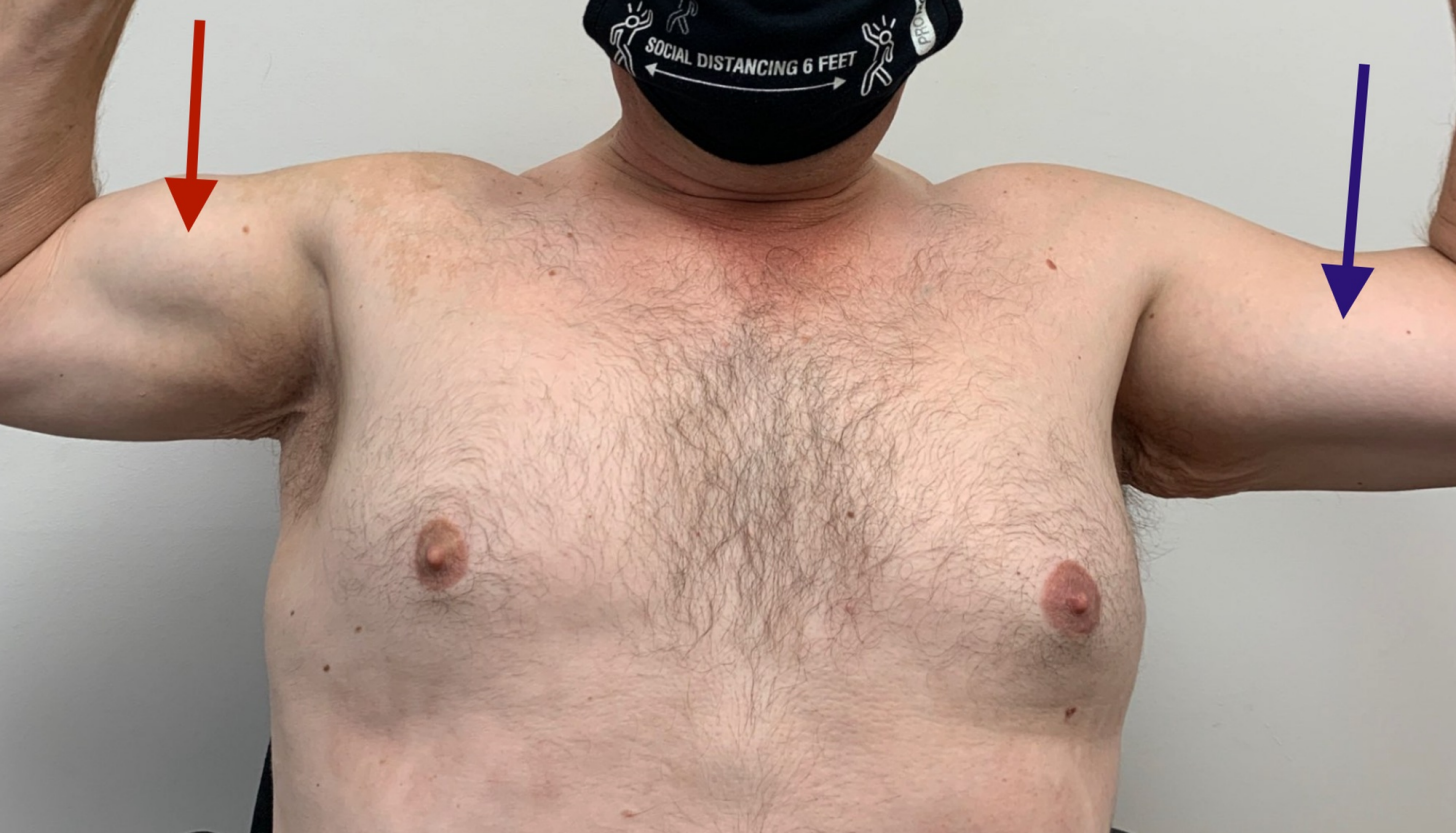
Demonstration of biceps atrophy: normal right biceps bulk (red arrow), obvious left biceps atrophy (blue arrow)

Left biceps atrophy is conspicuously demonstrated (Figure 1).

Deep tendon reflexes are graded using the standard scaling scale from 0-4. All the reflexes were lively except the left biceps which was conspicuously absent. Sensory examination revealed diminished pin prick over the lateral aspect of the forearm over the volar and extensor aspect.

**Figure 2:**
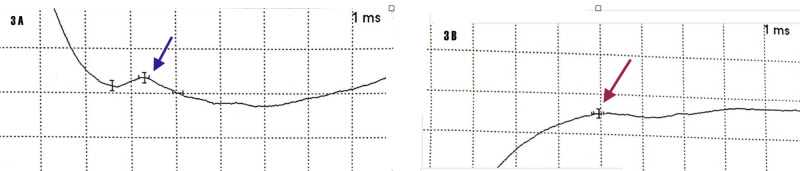
Nerve Conduction Study: 3A - normal right musculocutaneous sensory amplitude (blue arrow). 3B - absent left musculocutaneous sensory amplitude (red arrow); abscissa millisecond (ms), time base NCS: Nerve Conduction Study

We have derived a clinical phenotype: weakness of biceps, brachialis and coracobrachialis with sensory hypesthesia to pin prick in the distribution of the lateral musculocutaneous sensory nerve. We can confidently state that we have a musculocutaneous neuropathy. However, examinations are subjective, despite the fact that we have a pattern of recognition and striking left biceps atrophy with absent biceps reflex. The next step is to confirm these findings with a nerve conduction study/electromyogram. Indeed, we demonstrated an absent left musculocutaneous sensory nerve action potential and compared it to the contralateral side (Figure 2).

**Figure 3:**
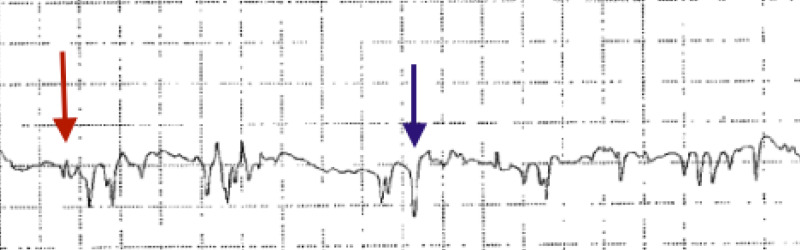
Electromyogram of left biceps muscle: florid fibrillation potentials (red arrow) and positive sharp waves (blue arrow) indicating active denervation of muscle

An electromyographic study of left upper extremity showed florid acute denervation of left biceps muscle (Figure 3).

**Figure 4:**
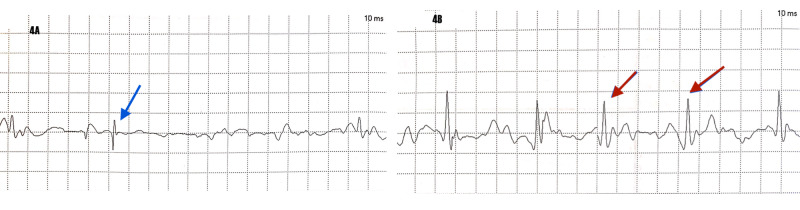
Electromyogram of left brachialis. 4A: fibrillation potentials (blue arrow) indicating acute denervation. 4B: rapidly firing motor units indicating motor neuron drop-out (red arrow); time base 10 milliseconds (ms) EMG: Electromyogram

The less involved left brachialis muscle was also studied by electromyography, and this revealed acute denervation with motor unit drop-out as demonstrated by rapidly firing motor units (Figure 4).

We have demonstrated acute denervation of the left biceps and left brachialis. The coracobrachialis muscle was not studied. All other muscle groups including the left supraspinatus, deltoids, triceps, brachioradialis, pronator teres, supinator, extensor carpi radialis longus, first dorsal interosseus and abductor pollicis brevis showed no evidence of denervation. With an absent left musculocutaneous sensory amplitude, we arrive at the diagnosis of a left musculocutaneous neuropathy.

**Figure 5:**
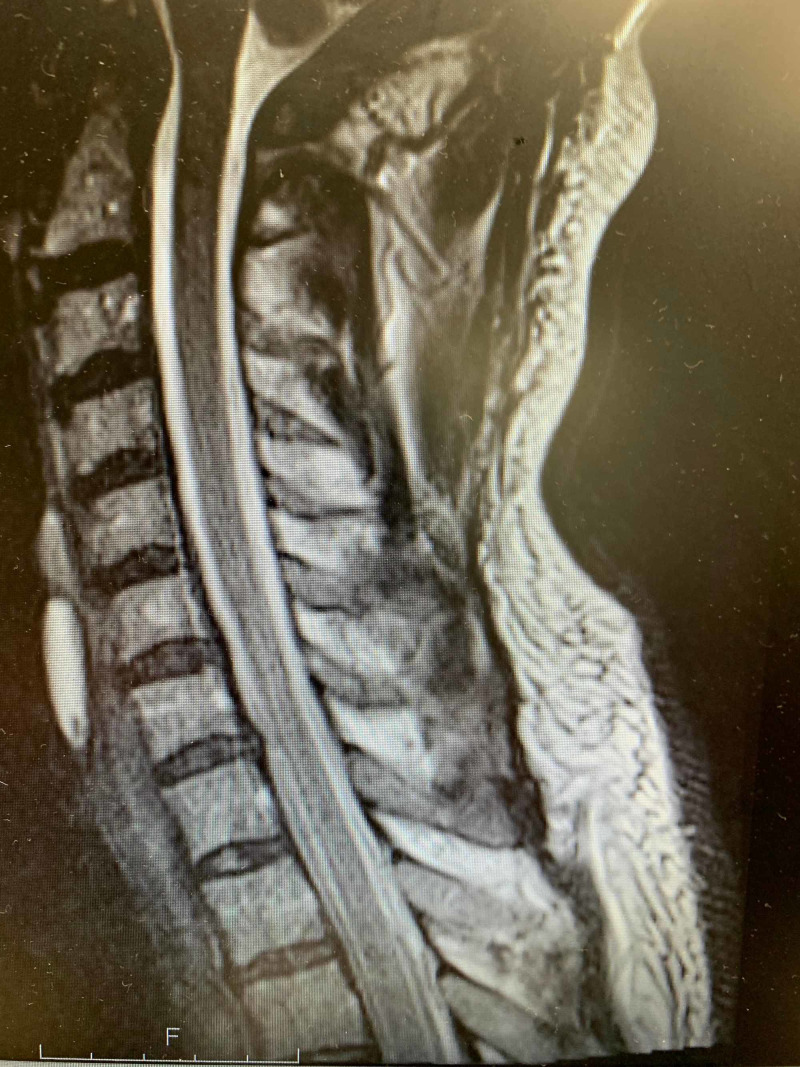
T2 weighted sagittal MRI: mild spondylosis; not enough to explain clinical findings MRI: Magnetic Resonance Imaging

A magnetic resonance image (MRI) of the cervical spine showed mild spondylosis (Figure 5). 

We believe this is most likely immune-mediated as the clinical profile is typical for a neuritis. It is unlikely to be a diabetic brachial plexopathy, which is also autoimmune, as his glucose levels are normal and his hemoglobin A1c is 6.2 (< 7) throughout his illness. There is a possibility that secukinumab, the anti-interleukin 17 biologic for the psoriatic arthritis, is the causative agent. Hence this was discontinued and his rheumatologist suggested many other possibilities for the treatment of his psoriatic arthritis. We conclude that the most likely possibility is an idiopathic immune-mediated brachial plexopathy affecting the MCN. The patient received intravenous solumedrol 500 milligram (mg) weekly for four weeks. The plan is to chase this with intravenous immunoglobulin should he not show any improvement. At six months, we plan to do magnetic resonance image of the brachial plexus and musculocutaneous nerve to look for hourglass constrictions.

## Discussion

Tsairis et al. studied the outcome of 84 patients with immune-mediated brachial plexopathy. They note that the upper, the lower, or the entire brachial plexus may be involved and it may be bilateral. The overall prognosis is excellent despite the severity and extent of the lesion. Their findings include that prior immunization does not alter the natural history of the disease. Whereas recovery may begin by one or two months, complete recovery may take up to three years [9]. 

In a study of 199 patients with immune-mediated brachial plexopathy, involvement of the upper and middle trunk with involvement of the long thoracic and/or suprascapular nerve occurred most frequently (71.1%). Recurrent attacks were found in 26.1% of the patients monitored over six years. Of note the middle and lower trunks of the brachial plexus were involved more frequently in women than men (23.1% versus 10.5%), and the functional outcome was worse in women [10]. 

The study of Cruz-Martinez et al. looked at 40 patients. Antecedent viral illness was seen in 22 cases. Pain of sudden onset was always the initial symptom, followed by weakness, mostly involving the shoulder girdle and bilateral in seven cases. Seven cases were also recurrent. Unusual patterns of weakness, such as facial and glossopharyngeal cranial nerves, phrenic nerve, and lateral antebrachial cutaneous nerve involvement were also recorded. They also found a descending frequency of involvement of the suprascapular, axillary, musculocutaneous, long thoracic, and radial nerves in this order [7].

The phenotypic patterns of weakness with diagnostic clues as seen in the immune-mediated brachial plexopathies are listed below (Table 1).

**Table 2:** Patterns of weakness of immune-mediated brachial plexopathies: The patterns listed are not mutually exclusive; combinations such as an upper trunk plexopathy with either a suprascapular nerve or long thoracic nerve involvement do occur.

STUDY	NERVE TRUNK	PATTERN OF WEAKNESS / LIST OF MOST AFFECTED MUSCLES	PATTERN RECOGNITION " SPOT DIAGNOSIS "
Cruz-Martinez A et al., 2017, [7]	Upper trunk of brachial plexus	Deltoids, supraspinatus, infraspinatus, biceps, brachioradialis	Shoulder girdle weakness
Alhammad RM et al., 2017, [11]	Lower trunk of brachial plexus	Ulnar nerve innervated muscles: flexor carpi ulnaris, sublimis, flexor digitorum profundus to ring and little fingers, interossei, adductor pollicis, abductor digit minimi Median nerve innervated muscles: abductor pollicis brevis, flexor pollicis longus, flexor digitorum profundus to index and middle fingers Radial nerve innervated muscles: extensor indicis proprius and extensor pollicis brevis	Weak hand, cannot form " O " sign
Zanette G et al., 2020, [5]. Hussey AJ et al., 2007, [12]	Musculocutaneous nerve	Biceps, brachialis, coracobrachialis	Flat biceps
Fransz DP et al., 2014, [13]	Long thoracic nerve	Serratus anterior	Winging of scapula
Odell JA, 2011, [14]	Phrenic nerve	Diaphragm	Elevated hemi-diaphragm, rarely bilateral
Cruz-Martinez A et al., 2017, [7]	Axillary nerve	Deltoids, teres minor, triceps	Weakness of shoulder elevation and elbow extension
Cruz-Martinez A et al., 2017, [7]	Suprascapular nerve	Supraspinatus, infraspinatus	Suprascapular atrophy
Renneis GD et al., 1980, [15]	Anterior interosseus nerve	Flexor pollicis longus, pronator quadratus, flexor digitorum profundus to index and middle finger	Cannot form " O " sign
Yang JS et al., 2015, [16]	Posterior interosseus nerve	All the muscles of the extensor compartment of the forearm except aconeus, brachioradialis and extensor carpi radialis longus	Finger drop: " Texas longhorn horn " sign

MRI and high-resolution ultrasound have revealed nerve trunk pathologies most notably pathognomic hourglass-like constrictions. These new findings have opened up a new method for more accurate diagnosis through high-resolution imaging [17]. 

Pan et al. report on five patients with immune-mediated brachial plexopathy with hourglass constriction in the affected nerves. The hourglass constrictions were in nerves outside the brachial plexus. Hourglass constrictions were identified in the severely affected nerves that had no response to treatment. The nerves were treated with neurolysis, resection and neurorrhaphy and resection with nerve graft. Neurolysis was performed at the sites of constrictions in two radial nerves and one median nerve. All nerves treated with external neurolysis achieved full recovery. Two nerves were treated with resection and neurorrhaphy. One attained full recovery and the other had a partial recovery. Of the three nerves treated with resection and nerve graft, one attained full recovery and two had incomplete recovery [18].

Suarez et al. performed brachial plexus biopsies in four patients. Conspicuous mononuclear inflammatory infiltrates were observed surrounding epineurial and endoneurial vessels, but without mural fibrinoid necrosis characteristic of necrotizing vasculitis. The infiltrates were composed of T- lymphocytes and contained a reactive germinal center with small cleaved and large non-cleaved (cluster of differentiation) CD20-positive B-lymphocytes in one patient. Another patient demonstrated a lymphocytic infiltrate around the soma of a spinal ganglion neuron. One patient's biopsy showed demyelinated axons and onion bulb formation [8].

Complement-fixing antibodies to peripheral nerve myelin (anti-PNM antibodies) and terminal complement activation products were reported to be increased in serum of patients with brachial plexus neuropathy compared with normal controls [19].

Zanette et al. report imaging findings in two cases of immune-mediated brachial plexopathy with selective brachialis muscle atrophy and fascicular involvement of the MCN [5]. There are a few other anecdotal reports among others [11,20]. Our case report adds to the existing literature. In particular we focus on the patterns of weakness in immune-mediated brachial plexopathies. These patterns are an exercise in anatomic localization and pattern recognition, and are useful in the diagnostic work-up, as brachial plexopathies are at times quite difficult to localize and frequently require NCS/EMG studies to help with the diagnosis. We further highlight the immune nature of this group of disorders. Lastly, we expand on the recent spectacular findings on MRI studies, which reveal the hourglass constrictions of peripheral nerves outside of the brachial plexus. This opens up a surgical window of opportunity for neurolysis in previously refractory cases.

## Conclusions

We describe the rare case of a musculocutaneous neuropathy arising from an immune-mediated brachial plexopathy and segue into the clinical semiology and pathophysiology of the immune-mediated brachial plexopathies. Clinical diagnostic strategies involve both deductive reasoning and pattern recognition. Brachial plexopathies are notoriously difficult to localize anatomically. In this article we summarize both the low and high-frequency patterns of weakness of the immune-mediated plexopathies. Unfortunately, the combinations of involvement of various muscle groups can be daunting especially when different parts of the brachial plexus are involved in a patchy manner. However, there are a few patterns that are easy to recognize, the so-called "spot diagnosis ". We highlight these patterns, being fully aware that this does not obviate the need for a thorough history taking and examination. We also dive into the pathology of the immune-mediated plexopathies. However, our account is laser-focused on one of the first histologic studies. Due to the scope of this article, we do not highlight the plethora of anecdotal reports on post-vaccination, post-viral, diabetic and biologic therapy-induced immune-mediated brachial plexopathies. What we highlight are the dramatic new radiologic findings on the hourglass constrictions of peripheral nerves in afflicted patients, with the new exciting opportunity for surgical decompression.

## References

[1] Reboucas F, Filho RB, Filardis C (2015). Anatomical study of the musculocutaneous nerve in relation to the coracoid process. Rev Bras Ortop.

[2] Mastaglia F (1986). Musculocutaneous neuropathy after strenuous physical activity. Med J Aust.

[3] Davidson JJ, Bassett III FJ, Nunley II J (1998). Musculocutaneous nerve entrapment revisited. J Shoulder Elbow Surg.

[4] Liveson JA (1984). Nerve lesions associated with shoulder dislocation; an electrodiagnostic study of 11 cases. J Neurol Neurosurg Psychiatry.

[5] Zanette G, Rasera A, Tamburin S (2020). Selective atrophy of the brachialis muscle in neuralgic amyotrophy: ultrasound imaging of fascicular nerve damage. J Neurol Neurosurg Psychiatry.

[6] Juel VC, Kiely JM, Leone KV (2000). Isolated musculocutaneous neuropathy caused by a proximal humeral exostosis. Neurology.

[7] Cruz-Martinez A, Barrio M, Arpa J (2002). Neuralgic amyotrophy: variable expression in 40 patients. J Peripher Nerv Syst.

[8] Suarez GA, Giannini C, Bosch EP (1996). Immune brachial plexus neuropathy: suggestive evidence for an inflammatory-immune pathogenesis. Neurology.

[9] Tsairis P, Dyck PJ, Mulder DW (1972). Natural history of brachial plexus neuropathy: report on 99 patients. Arch Neurol.

[10] van Alfen N, van Engelen B (2006). The clinical spectrum of neuralgic amyotrophy in 246 cases. Brain.

[11] Alhammad RM, Dronca RS, Kottschade LA (2017). Brachial plexus neuritis associated with anti-programmed cell death-1 antibodies: report of 2 cases. Mayo Clin Proc Innov Qual Outcomes.

[12] Hussey AJ, O'Brien CP, Regan PJ (2007). Parsonage-Turner syndrome—case report and literature review. Hand.

[13] Fransz DP, Schonhuth CP, Postma TJ (2014). Parsonage-Turner syndrome following post-exposure prophylaxis. BMC Musculoskelet Disord.

[14] Odell JA, Kennelly K, Stauffer J (2011). Phrenic nerve palsy and Parsonage-Turner syndrome. Ann Thorac Surg.

[15] Renneis GD, Ochoa J (1980). Neuralgia amyotrophy manifesting as anterior interosseous nerve palsy. Muscle Nerve.

[16] Yang JS, Cho YJ, Kang HS (2015). Neuralgic amyotrophy manifesting as mimicking posterior interosseous nerve palsy. J Korean Neurosurg Soc.

[17] Gstoettner C, Mayer JA, Rassam S (2020). Neuralgic amyotrophy: a paradigm shift in diagnosis and treatment. J Neurol Neurosurg Psychiatry.

[18] Pan YW, Wang S, Tian G (2011). Typical brachial neuritis (Parsonage-Turner syndrome) with hourglass-like constrictions in the affected nerves. J Hand Surg Am.

[19] Vriesendorp FJ, Dmytrenko GS, Dietrich T (1993). Anti-peripheral nerve myelin antibodies and terminal activation products of complement in serum of patients with acute brachial plexus neuropathy. Arch Neurol.

[20] Besleaga D, Castellano V, Lutz C (2010). Musculocutaneous neuropathy: case report and discussion. HSS J.

